# Supplementary material for: Background malaria incidence and parasitemia during the three-dose RTS,S/AS01 vaccination series do not reduce magnitude of antibody response nor efficacy against the first case of malaria
Source: BMC Infect Dis. 2023 Oct 23;23:716. doi: 10.1186/s12879-023-08699-7 (PMC10594884; doi:10.1186/s12879-023-08699-7)
Supplement: Supplementary file 1 — Supplemental Figure 1: Distribution of MTI values within and across study sites using a) our original predicted values b) Plasmodium falciparum incidence rate and c) Plasmodium falciparum infection prevalence among 2-10 year olds. Supplemental Figure 2: Anti-CSP Antibody Response by Study Area (1 Month Post-Vaccination). Supplemental Figure 3: Anti-CSP Antibody Response by Transmission Intensity, All Study Areas and Study Area Adjusted (A: 1 Month Pre-Vaccination, B: 1 Month Post-Vaccination) using Plasmodium falciparum incidence rate per person per year. Supplemental Figure 4: Anti-CSP Antibody Response by Transmission Intensity, All Study Areas and Study Area Adjusted (A: 1 Month Pre-Vaccination, B: 1 Month Post-Vaccination) using Plasmodium falciparum infection prevalence among 2-10 year olds. Supplemental Figure 5: Efficacy Against the First Case of Malaria Over Time, Transmission Intensity, and Cases During Vaccination using Plasmodium falciparum incidence rate. Supplemental Figure 6: Multiplier Effect of RTS,S/AS01 Vaccination on the Time to First Case of Malaria A higher multiplier implies a greater delay until the first case of malaria (e.g. a multiplier of 2 where the control group experiences their first case of malaria in 100 days will extend the time to the first case to 200 days). The null value is 1. Supplemental Figure 7: Efficacy Against the First Case of Malaria Over Time, Transmission Intensity, and Cases During Vaccination, Stratified by Study Area. [file 12879_2023_8699_MOESM1_ESM.docx]

**Supplement**

**Sensitivity Analysis of Malaria Transmission Intensity**

To assess the robustness of our predicted Malaria Transmission Intensity (MTI) estimates, we compared our estimates with publicly available data on malaria incidence from the Malaria Atlas Project (MAP) using the malariaAtlas package in R.^1^ MAP is a widely used data platform that combines malaria data from Demographic Health Surveys (DHS), Malaria Indicator Cluster Surveys (MICS), government reports, and researcher contributions with cutting-edge geospatial modeling to provide global estimates of malaria risk and burden at a spatial resolution of 5km by 5km.^2^ We extracted two values of MTI using the MAP data: The *Plasmodium falciparum* Incidence Rate, defined as the number of newly diagnosed *Plasmodium falciparum* cases per person in a given year, and the Infection Prevalence (*Pf*Pr), defined as the Proportion of Children 2 to 10 years of age showing detectable *Plasmodium falciparum* parasite during a given year. Both measures were extracted for the years 2009 to 2014 and averaged for each location to yield a mean MTI over the study period.

The comparisons of our predicted MTI estimates with the MAP estimates are shown below (Supplemental Figure 1). Our predicted cases per person per year were higher as compared to the MAP estimates likely due to differences in our denominator calculations where we restricted our denominator to infants and children as compared to the whole population denominator used in MAP. Importantly though, we notice that the relative distributions of incidence rate within and across the three sites is similar with Lambaréné, Gabon having the lowest MTI while Kintampo, Ghana has the highest. Additionally, the *Plasmodium falciparum* prevalence within 2-to-10-year-olds - a measure of malaria transmission intensity in areas with no specific data - also exhibits a similar violin plot distribution compared to our predicted violin plots.

**Impact on antibody response and RTS,S/AS01 vaccine efficacy against the first case of malaria**

Supplemental Figure 2 shows a boxplot of antibody levels 1-month post-vaccination with the third dose of RTS,S/AS01. Supplemental Figures 3 and 4 display the relationship between Anti-CSP response and malaria transmission intensity, defined by MAP mean incidence per person year and mean malaria infection prevalence, respectively. These two measures of transmission intensity provided very similar results.

One month before vaccination with RTS,S/AS01 or a control vaccine, participant anti-CSP levels were essentially zero, regardless of malaria transmission intensity. One month after the third dose, those who received the control vaccine maintained low antibody levels, regardless of malaria transmission intensity, while those who received the three-dose RTS,S/AS01 vaccine series had greatly increased antibody levels. Among those who received RTS,S/AS01, antibody level was positively associated with transmission intensity, both overall and within study areas.

Supplemental Figure 5 highlights the relationship between vaccine efficacy and malaria transmission intensity (MAP mean incidence) and cases during vaccination using a Cox proportional hazards model. Similar to our main analysis, we observed that while RTS,S/AS01 efficacy waned over time and that there was very weak evidence that efficacy against the first case of malaria varied by transmission intensity or parasitemia during the three-dose vaccination series.

**
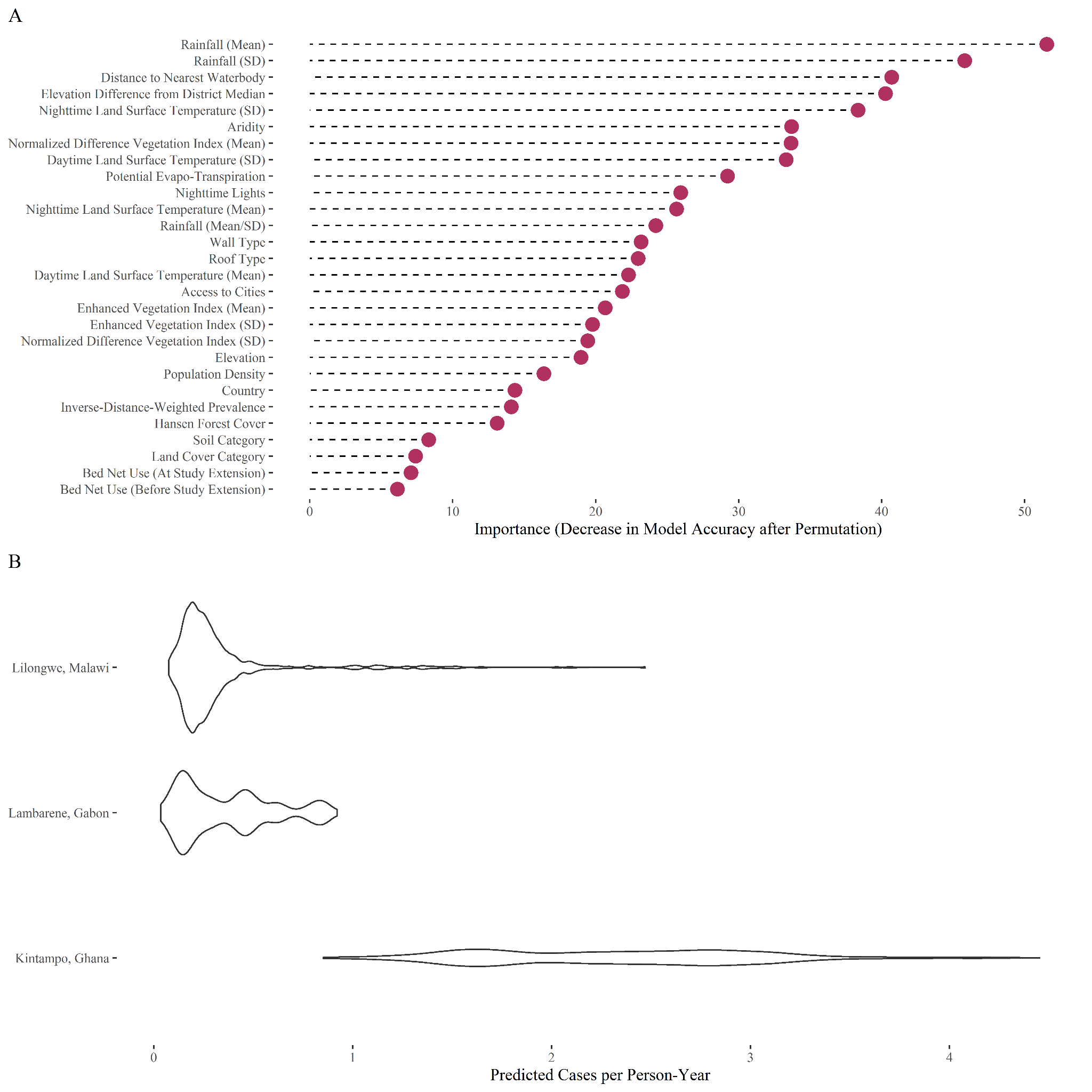

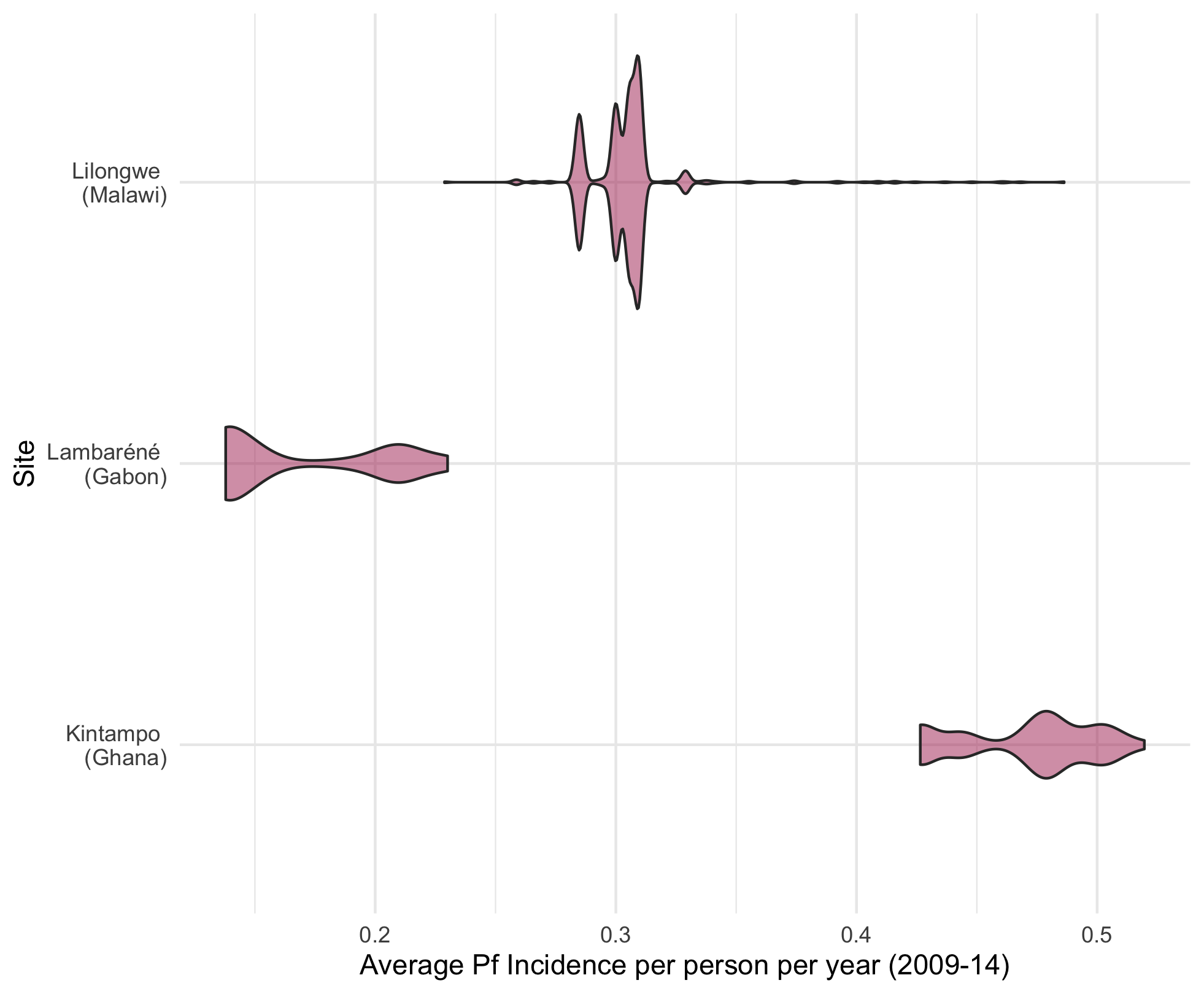
**

**
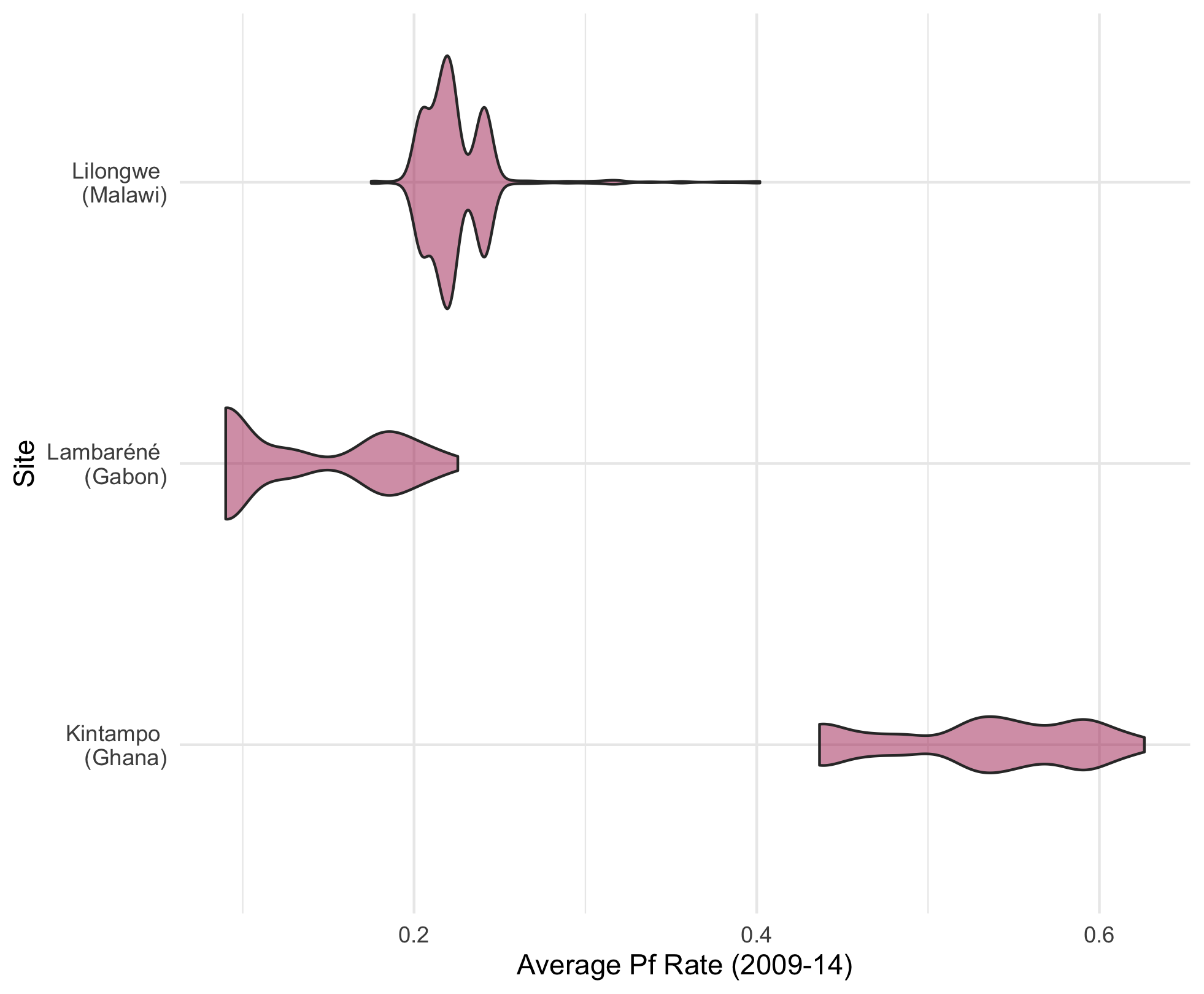
**

**Supplemental Figure 1: Distribution of MTI values within and across study sites using a) our original predicted values b) *Plasmodium falciparum* incidence rate and c) *Plasmodium falciparum* infection prevalence among 2-10 year olds**

**
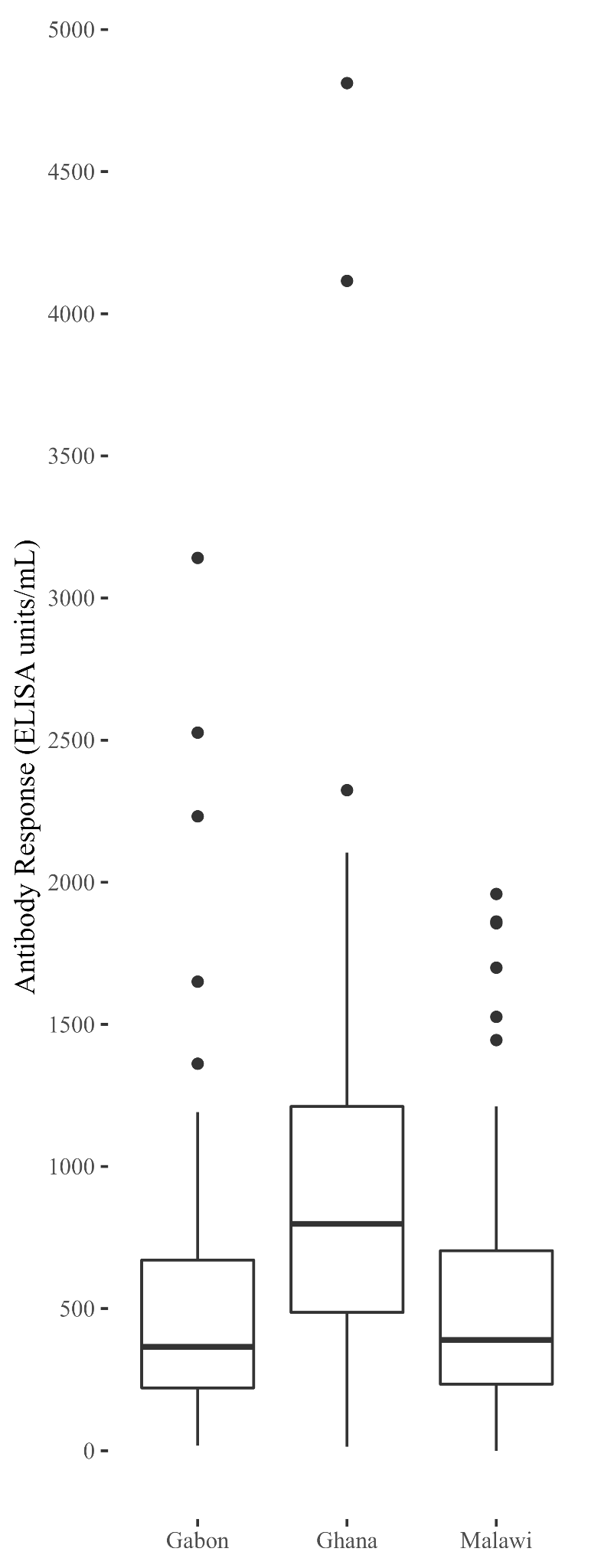
**

**Supplemental Figure 2: Anti-CSP Antibody Response by Study Area (1 Month Post-Vaccination)**

**
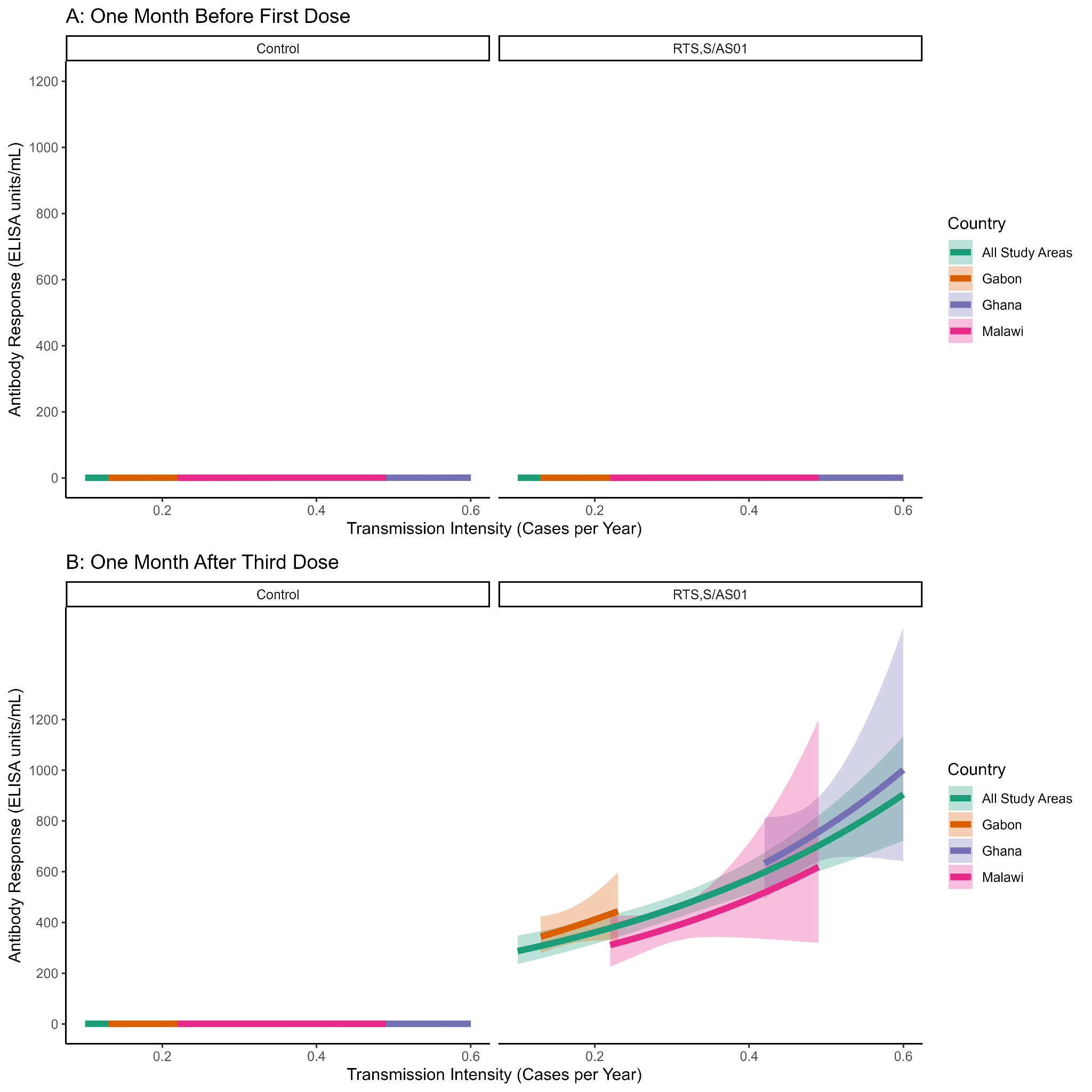
**

**Supplemental Figure 3: Anti-CSP Antibody Response by Transmission Intensity, All Study Areas and Study Area Adjusted (A: 1 Month Pre-Vaccination, B: 1 Month Post-Vaccination) using *Plasmodium falciparum* incidence rate per person per year.**

**
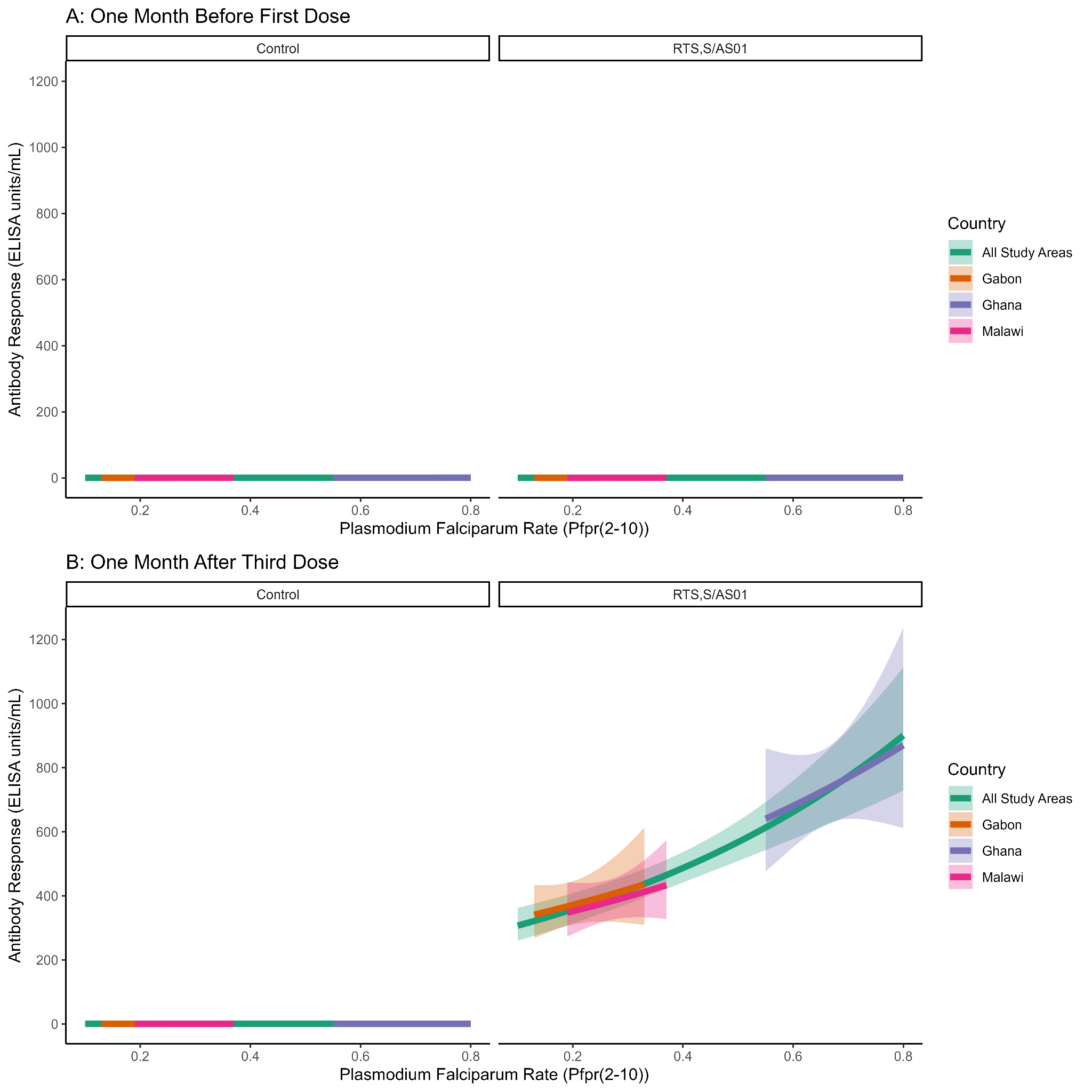
Supplemental Figure 4: Anti-CSP Antibody Response by Transmission Intensity, All Study Areas and Study Area Adjusted (A: 1 Month Pre-Vaccination, B: 1 Month Post-Vaccination) using *Plasmodium falciparum* infection prevalence among 2-10 year olds.**

**
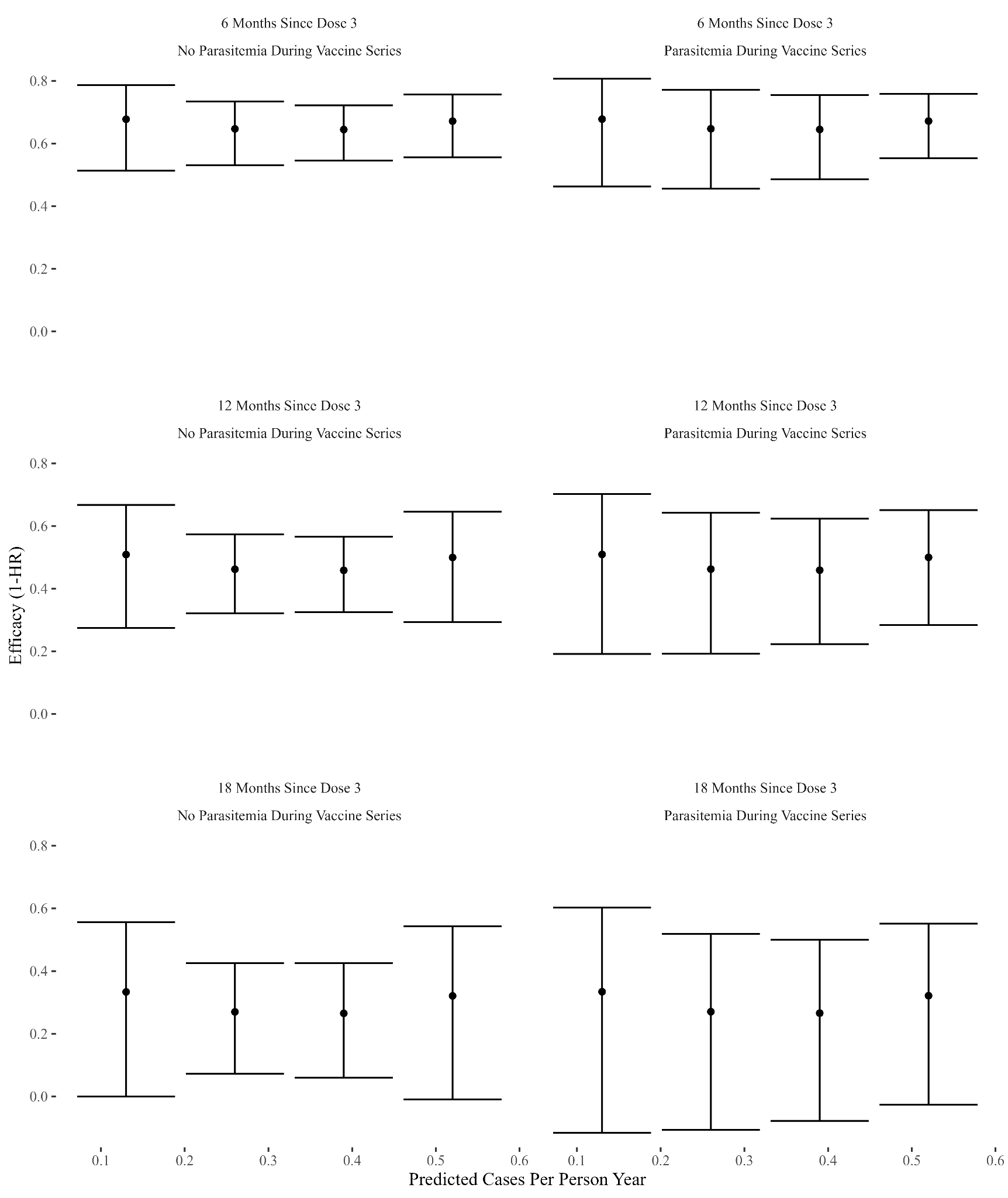
**

**Supplemental Figure 5: Efficacy Against the First Case of Malaria Over Time, Transmission Intensity, and Cases During Vaccination using *Plasmodium falciparum* incidence rate**

**
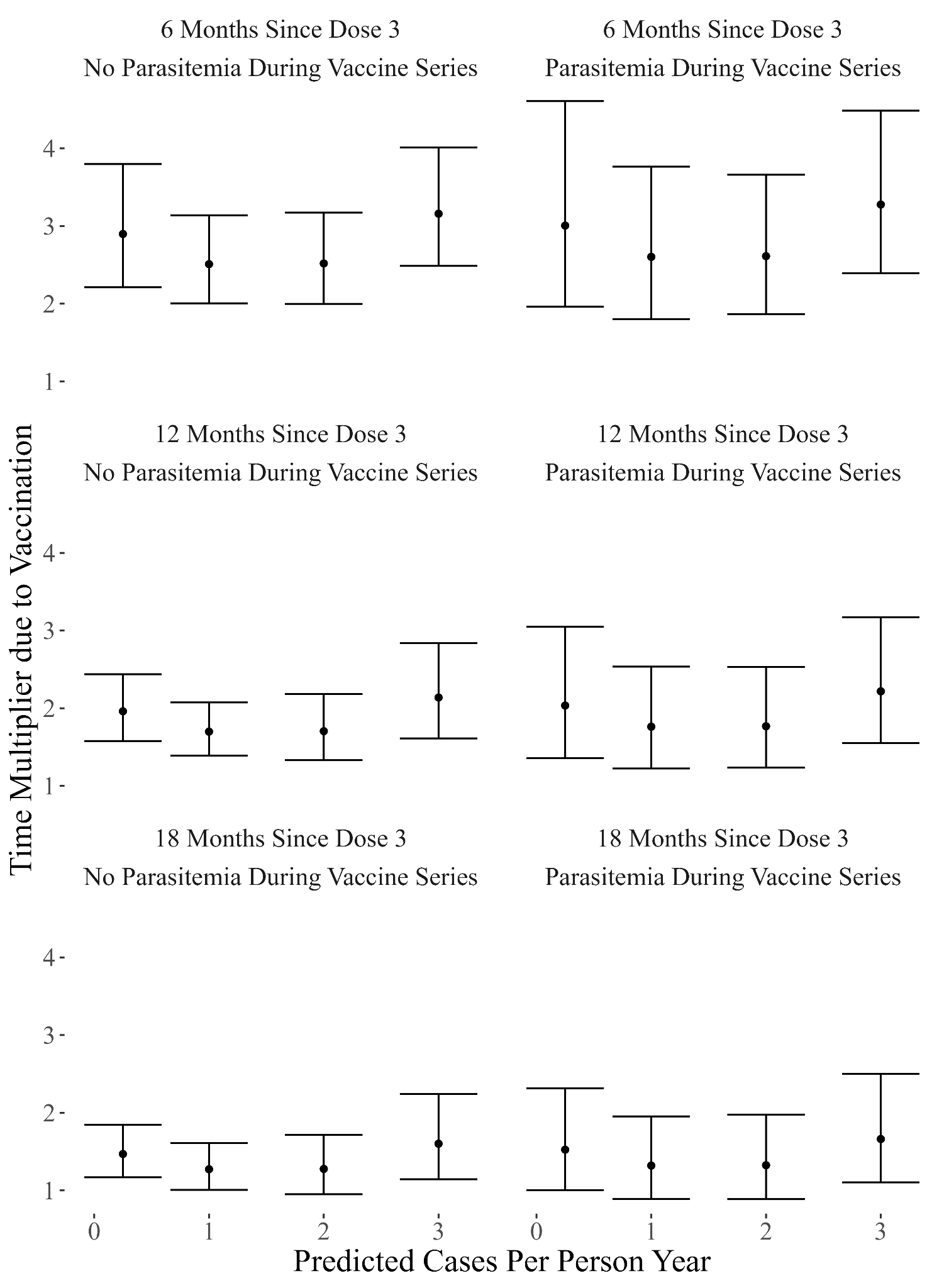
**

**Supplemental Figure 6: Multiplier Effect of RTS,S/AS01 Vaccination on the Time to First Case of Malaria**

*A higher multiplier implies a greater delay until the first case of malaria (e.g. a multiplier of 2 where the control group experiences their first case of malaria in 100 days will extend the time to the first case to 200 days). The null value is 1.*

**
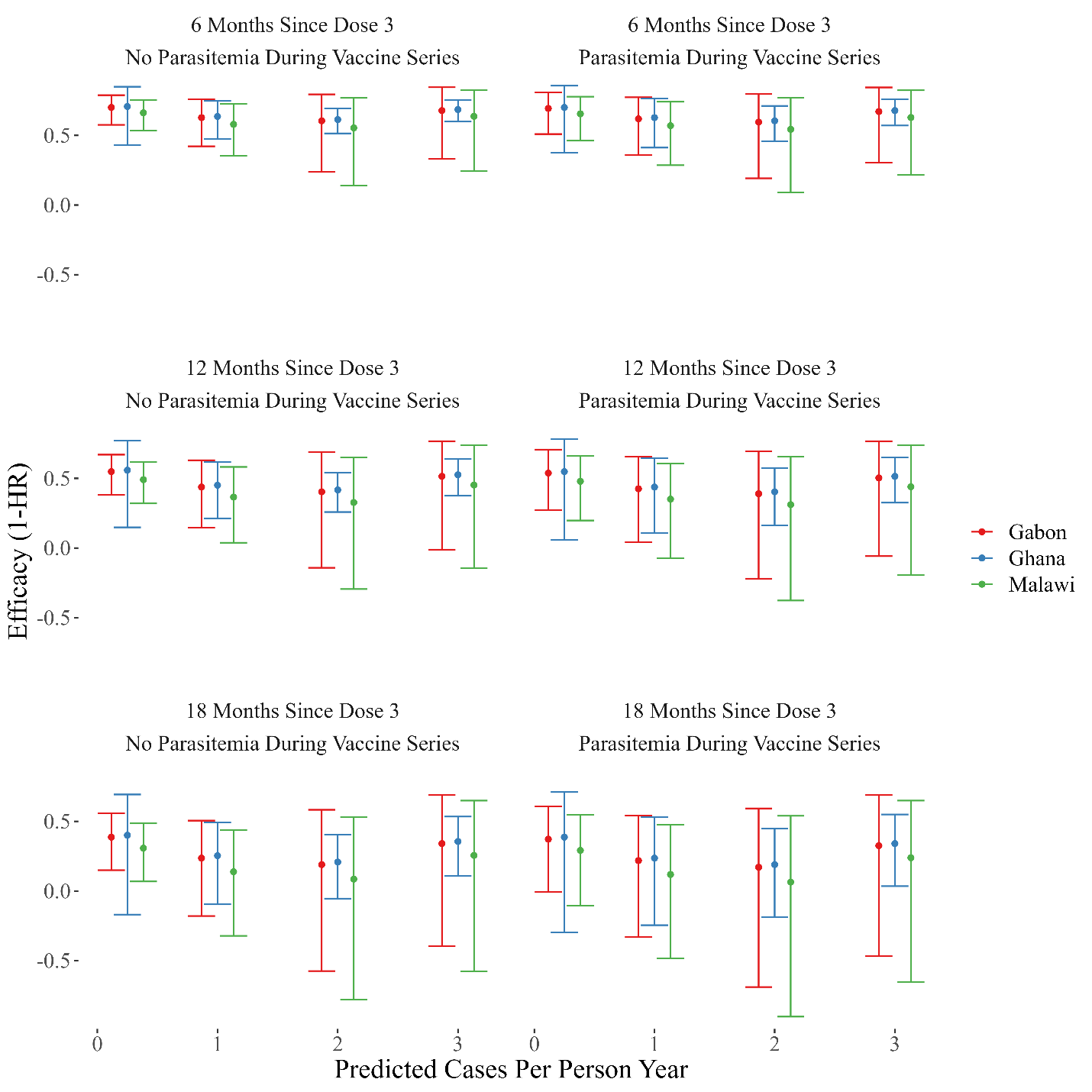
Supplemental Figure 7: Efficacy Against the First Case of Malaria Over Time, Transmission Intensity, and Cases During Vaccination, Stratified by Study Area**

**References**

1 Pfeffer DA, Lucas TCD, May D, *et al.* malariaAtlas: an R interface to global malariometric data hosted by the Malaria Atlas Project. *Malar J* 2018; **17**: 352.

2 Hay SI, Snow RW. The malaria Atlas Project: developing global maps of malaria risk. *PLoS Med* 2006; **3**: e473.
